# The Peptidoglycan Biosynthesis Gene *murC* in *Frankia:* Actinorhizal vs. Plant Type

**DOI:** 10.3390/genes11040432

**Published:** 2020-04-16

**Authors:** Fede Berckx, Daniel Wibberg, Jörn Kalinowski, Katharina Pawlowski

**Affiliations:** 1 Department of Ecology, Environment, and Plant Sciences, Stockholm University, 10691 Stockholm, Sweden; fede.berckx@su.se; 2 Center for Biotechnology (CeBiTec), Bielefeld University, 33615 Bielefeld, Germany; dwibberg@cebitec.uni-bielefeld.de (D.W.); joern@CeBiTec.Uni-Bielefeld.DE (J.K.)

**Keywords:** Peptidoglycan, *MurC*, *Frankia*, nitrogen fixation, actinorhizal symbiosis, root nodules

## Abstract

Nitrogen-fixing Actinobacteria of the genus *Frankia* can be subdivided into four phylogenetically distinct clades; members of clusters one to three engage in nitrogen-fixing root nodule symbioses with actinorhizal plants. Mur enzymes are responsible for the biosynthesis of the peptidoglycan layer of bacteria. The four *Mur ligases,* *MurC*, *MurD*, *MurE*, and *MurF*, catalyse the addition of a short polypeptide to UDP-N-acetylmuramic acid. *Frankia* strains of cluster-2 and cluster-3 contain two copies of *murC,* while the strains of cluster-1 and cluster-4 contain only one. Phylogenetically, the protein encoded by the *murC* gene shared only by cluster-2 and cluster-3, termed *MurC1*, groups with *MurC* proteins of other Actinobacteria. The protein encoded by the *murC* gene found in all *Frankia* strains, *MurC2,* shows a higher similarity to the *MurC* proteins of plants than of Actinobacteria*. MurC2* could have been either acquired via horizontal gene transfer or via gene duplication and convergent evolution, while *murC1* was subsequently lost in the cluster-1 and cluster-4 strains. In the nodules induced by the cluster-2 strains, the expression levels of *murC2* were significantly higher than those of *murC1.* Thus, there is clear sequence divergence between both types of *Frankia MurC*, and *Frankia murC1* is in the process of being replaced by *murC2*, indicating selection in favour of *murC2*. Nevertheless, protein modelling showed no major structural differences between the *MurCs* from any phylogenetic group examined.

## 1. Introduction

Bacteria are exposed to various environmental stresses against which the cell wall has to provide protection, while also maintaining the cell shape. Intracellular symbiotic bacteria need to survive both inside and outside the host, which can create the need for cell wall modifications. The cell walls of Gram-positive bacteria are characterized by a ca. 30 nm thick peptidoglycan (PG) layer. As a much thinner layer in the cell wall, PG can also be found in Gram-negative bacteria (3–6 nm), cyanobacteria (10 nm), and moss chloroplasts (5 nm) [1,2,3,4]. PG is synthesized by the activity of Mur ligases, which add a number of amino acids to UDP-N-acetylmuramic acid to connect different strands. In a series of ATP-dependent reactions, L-alanine (*MurC*), D-glutamate (*MurD*), L-lysine (*MurE*), and D-alanyl-D-alanine (*MurF*) are added sequentially. The precise sequence of the reactions catalysed by these enzymes was reviewed by Barreteau et al. [5] and tested in *Mycobacterium tuberculosis* [6], as they represent an interesting target for antibiotic drug discovery. Several reports on the activity of Mur ligases have been published for non-pathogenic bacteria. In the cyanobacterium *Anabena* sp., *murC* and *murB* play an important role in heterocyst differentiation [7]. Although, so far, PG layers have not been identified for the chloroplasts of Spermatophyta, it is known that *Arabidopsis thaliana* contains a copy of *murE,* and the mutation of this gene leads to the reduced chlorophyll content of leaves, indicating an essential role of *murE* for chloroplast development [8]. Yet, a *murE* mutant of the moss *Physcomitrella patens* could be complemented with a cyanobacterial *murE* gene, but not with Arabidopsis *murE,* indicating functional divergence. At any rate, the occurrence of Mur ligase genes in diverse eukaryote genomes from green algae to angiosperms have made them the subject of study into plastid development, endosymbiosis, and the multiple events of horizontal gene transfer (HGT) that led to the evolution of land plants.

*Frankia* is a genus of nitrogen-fixing soil actinobacteria. They engage in root nodule endosymbiosis with a diverse group of host plants within the orders of the Fagales, Rosales, and Cucurbitales, collectively referred to as actinorhizal plants (reviewed by Pawlowski and Demchenko [9]). The genus can be split into four phylogenetically distinct clades, known as cluster-1 to cluster-4; the first three clusters contain the symbiotic strains [10]. The phylogenetic relationships between the clusters has been a point of discussion based on use of single marker genes [11,12], but recent studies using multiple phylogenetic markers or the core genome have shown that cluster-2 is the earliest divergent symbiotic clade [13,14,15,16]. Strains belonging to cluster-2 have a particular low saprotrophic potential. Within cluster two, so far, only two strains could be grown in culture, namely: *Frankia coriariae* BMG5.1 [17] and the closely related strain BMG5.30 [18]. Recent studies on the *Frankia* genome sequences isolated from whole nodules, or from *Frankia* vesicles isolated from nodules, have led to the elucidation of the phylogeny of cluster-2 and the identification of several lineages within this cluster [13,14,15]. Analysis of these genomes revealed that *murC,* encoding the enzyme that adds L-alanine as the first amino acid to UDP-N-actetylmuramic acid in the course of cell wall PG biosynthesis, was present in two divergent copies in all of the genomes of the *Frankia* cluster-2 strains available.

No gene duplication has been reported for *murC* thus far. Therefore, we were interested in the function and the origin of both *murC* copies. The presence of two copies of a given gene in a genome can be explained via two different scenarios. One scenario is gene duplication (reviewed by Hurles [19]), which can occur through non-homologous recombination or through retrotransposition. If the newly acquired copy of the gene becomes fixed, three options are possible. First, either one of the copies can lose its function and become a pseudogene. Second, one of the copies can gain a new function, a process referred to as neofunctionalization. Third, the gene function can be split over the two copies, referred to as subfunctionalization. In this case, normally, the two genes are expressed under different conditions. The other scenario to explain two copies of a given gene in the genome would be horizontal gene transfer (HGT). For example, the intracellular accommodation of cyanobacteria led to HGT events between the cyanobacteria and host, ultimately leading to the evolution of the plastid, although the origin of the plastid envelope is not yet fully understood [20]. In other words, the second copy of *murC* in *Frankia* could have been acquired through HGT, either from another soil bacterium or from the host plastid via the host nucleus.

This study aimed to answer the questions if one of the two *Frankia murC* copies originated from gene duplication or from HGT, and whether there is evidence for subfunctionalization. A thorough a phylogenetic analysis of *Frankia* MurC proteins revealed that the strains from cluster-2 and cluster-3 contained two types of MurC, MurC1 and MurC2, while the strains from cluster-1 and cluster-4 contained only MurC2. Protein structural modelling was performed for different types of *MurC* in order to analyse the structural conservation between the proteins, and the expression of the two versions of *murC* was studied in representatives of cluster-2 and cluster-3 using RT-qPCR. 

## 2. Materials and Methods 

### 2.1. Biological Material

The expression of *murC1* and *murC2* was analysed in the nodules of *Datisca glomerata* (C. Presl) Paill. induced by *Candidatus* Frankia californiensis Dg2 [21] and by *Candidatus* Frankia datiscae Cm1 [14], respectively. It was analysed in the nodules of *Coriaria nepalensis* Wall. induced by *Candiatus* Frankia datiscae Dg1 [15], in the nodules of *Coriaria arborea* Linds. induced by *Candidatus* Frankia meriodionalis Cppng1 [14], and in the nodules of *Coriaria myrtifolia* induced by *Frankia coriariae* BMG5.1 [17]. The expression was also analysed in the nodules of *Discaria trinervis* (Gillies ex hook.) Reiche induced by *Frankia discariae* BCU110501 [22]. Plants were germinated from seeds and grown in a greenhouse under a 16/8 h light/dark regime at 23/18 °C. Plants were supplied with a quarter strength Hoagland’s solution with 10 mM KNO_3_ [23] once a week, and with deionized water twice a week. Eight weeks after germination, the plants were inoculated with crushed nodules of *D. glomerate,* which were then used for the propagation of Dg1, Dg2, and Cm1, separately. Crushed nodules of *C. arborea* induced by Cppng1 [14] were used for inoculation with Cppng1. For inoculation with *F. coriariae* BMG5.1 [17] and *F. discariae* BCU110501 [22], in vitro cultures of *Frankia* were used, which had been grown in a BAP medium without nitrogen [24], adjusted to pH 9 for BMG5.1, at 28 °C for 35 and 28 d, respectively. For inoculation, the cells were pelleted and washed twice with sterile double-distilled H_2_O. At the time of inoculation, the washed root systems of the plants were evaluated to confirm that no plant was already nodulated. After inoculation, plants were supplied once per week with a quarter Hoagland’s medium without nitrogen, and twice a week with deionized water. Nodules of *D. glomerata* were harvested twelve weeks after inoculation, while nodules of *C. arborea, C. myrtifolia,* and of *D. trinervis* were harvested four months after inoculation. The nodules were frozen in liquid nitrogen and stored at −80 °C. 

Cultures of *F. discariae* BCU110501 grown in a BAP medium were used for the analysis of the expression levels of *murC1* and *murC2* in vitro. 

### 2.2. Gene Expression Analysis using Real-Time Quantitative PCR (RT-qPCR)

Primer pairs were designed for the consensus region of *murC1* and for the consensus region of *murC2* for Dg1, Dg2, and BMG5.1, while separate primer pairs were designed for Cppng1 and for BCU110501. All of the primers were designed using Primer3, available on Benchling with standard settings (Benchling Biology Software [25]); a list of all of the primers used in this study is provided in Supplementary Table S1. RNA isolation, gDNA digestion, cDNA synthesis, and RT-qPCR analysis were performed as described previously [14]. The gene encoding translation initiation factor three (IF-3), *infC,* was used as a reference gene to normalize the expression data [14,26]. An unpaired two-tailed Student’s *t*-test was used to test if the copies were expressed at significantly different levels. The statistical analysis and data visualisation were performed in RStudio (RStudio team [27]). 

### 2.3. Phylogenetic and Synteny Analysis

All of the *murC* gene sequences of plants and bacteria, publicly available in the NCBI GenBank (www.ncbi.nlm.nih.gov/genbank) database, were selected for the calculation of a phylogenetic tree. Amino acid sequences of both MurC1 and MurC2 were taken from *Frankia* cluster-2 strains and were used for identifying homologous sequences using blastP. *Frankia* protein sequences were taken from GenDB [28] and the JGI database [29]. Angiosperm and cyanobacterial sequences were taken from GenBank. Liverwort, hornwort, moss, fern, and conifer sequences were taken from the 1KP database (www.oneKP.com [30,31,32]). Additional bacterial sequences were taken from the UniProt database; here, only the reviewed sequences were selected. All of the sequences were used for a blastP search against the GenBank database to confirm MurC identity. All of the sequences used are given in Supplementary Table S2. 

First, multiple alignment for all protein sequences of the *murC* genes were created using the CLUSTAL Omega algorithm with 25 iterations and were trimmed using UGene [33,34]. The model of substitution was predicted using ModelTest-NG on the CIPRES portal [35]. The phylogenetic tree was constructed using RAxML on the CIPRES portal with predefined settings, using the LG model of amino acid evolution and with 250 bootstrap iterations [35].

The *murC* synteny in *Frankia* genomes was studied in the GenDB database for the genomes of cluster-2 strains, and in the JGI database for the genomes of cluster-1, -3, and -4 strains. 

### 2.4. Sequence Similarity Analysis

Based on the phylogenetic tree, the amino acid sequence data were split into seven major groups, as follows: Gram-positive bacteria (genera *Streptomyces, Thodococcus, Mycolicibacterium, Mycobacterium, Corneybacterium, Saccharopolyspora, Saccharamonospora,* and *Bifidobacterium*); *Frankia* MurC1, *Frankia* MurC2, plants (vascular plants and Bryophyta); *Chlamydia* and cyanobacteria (genera *Gloeomargarita, Thermosynechococcus, Cyanobacterium, Nostoc, Gloeothece, Synechococcus,* and *Prochlorococcus*); Gram-negative bacteria (genera *Goeobacter, Shewanella, Buchnera, Samonella, Vibrio, Pseuodomonas, Burkholderia, Rickettsia, Bradyrhizobium, Sinorhizobium, Rhizobium,* and *Mesorhizobium);* and a group containing the genera *Campylobacter, Helicobacter,* and *Thermotoga* (abbreviated TheCaHe). For each group, the consensus sequence was determined at a 75% similarity threshold using UGene, and a heatmap was visualized for the consensus sequences using the packages *seqir* and *heatmaply* in RStudio [27]. An interactive heatmap with all of the sequences used individually is given in Supplementary Figure S1.

### 2.5. Tetranucleotide Frequency

IslandViewer 4 [36] integrates genomic island predictions from multiple methods—IslandPath-DIMOB based on nucleotide bias and the presence of mobility genes, SIGI-HMM based on codon usage bias with a Hidden Markov Model approach, IslandPick based on a comparative genomics approach and the previously published Islander database, a fourth highly precise method that specifically predicts GIs integrated into tRNAs and tmRNAs with a precise definition of boundaries.

### 2.6. Protein Modelling

A MurC model was built for each group using SWISS-MODEL [37,38,39,40,41]. Because of the variation of sequences making up the outgroup, no protein was modelled for the TheCaHe group. The best model for each group was selected, and the different models were compared in 3D using the Dali server [42] and using the crystal structure of *Haemophilus influenzae* as a template (PDB id:1P31 [43]). The conservation within each group was visualised using Consurf [44,45,46], which was also used to compare the protein structure between the different groups. 

## 3. Results

### 3.1. Synteny Analysis Indicates Differences in Gene Organization of murC in the Four Frankia Clusters

**Figure 1:**
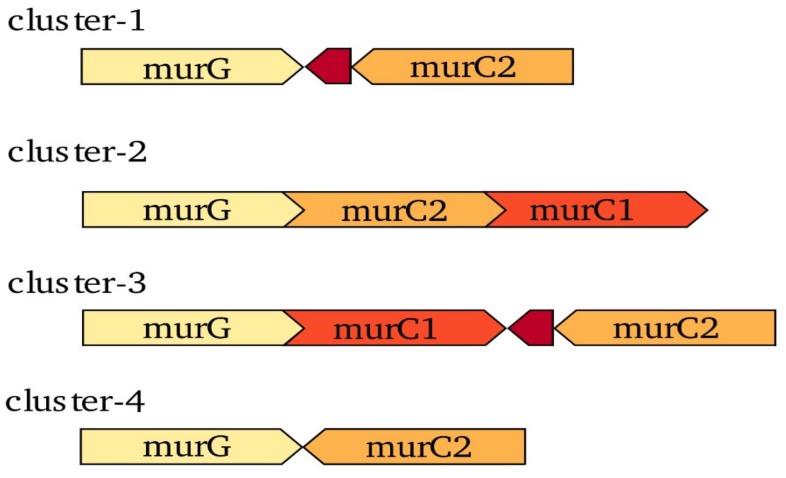
Gene neighbourhood of *murC* genes in the different *Frankia* clusters. Representatives of cluster-1 include *Frankia alni* ACN14a, *Frankia alni* AvcI1, and *Frankia casuarinae* CcI3. Cluster-2 representatives include *Candidatus* Frankia datiscae Dg1, *Candidatus* Frankia datiscae Cm1, *Candidatus* Frankia datiscae Cj1, *Candidatus* Frankia californiensis Dg2, *Candidatus* Frankia californiensis Cv1, *Candidatus* Frankia meridionalis Cppng1, *Frankia coriariae* BMG5.1, and *Frankia coriariae* BMG5.30. Cluster-3 representatives include *Frankia elaeagni* EAN1pec, *Frankia soli* NRRL_B16219, and *Frankia discariae* BCU0110501. Finally, cluster-4 representatives include *Frankia asymbiotica* NRRL B-16386, *Frankia inefficax* Eul1c, *Frankia saprophytica* CN3, and *Frankia* sp. DC12. *MurG,* labelled in light yellow, is used as a reference of the orientation of the different *murC* genes. *MurC1,* labelled in light red, was found in the strains from cluster-2 and cluster-3. *MurC2,* presented in orange, was found in all of the strains from all four clusters. A bioinformatically-predicted open reading frame encoding a nitroreductase family deazaflavin-dependent oxidoreductase, labelled in dark red, was found in cluster-1 and cluster-3. In clusters-1 and -4, *murG* is the last gene in a long operon of genes encoding the enzymes involved in peptidoglycan biosynthesis; in cluster-3, it is the second-to-last gene in this conserved operon. In cluster-2, *murG–murC2–murC1* alone form an operon.

Figure 1 shows the organisation of the different copies of *murC* in the genomes of *Frankia* strains from the four different clusters, with *murG* as a reference. Only the strains from cluster-2 and -3 contain two copies of *murC, murC1* and *murC2,* while the strains from cluster-1 and -4 contain only one copy, *murC2.* The organisation of the two copies differs in cluster-2 from that in cluster-3. While in the genomes of the cluster-2 strains, the two copies have the same orientation and might be organised in an operon together with *murG* (ca. 100 bp between *murC2* and *murC1),* in the genomes of the cluster-3 strains, *murC1* and *murC2* show opposite orientations, and a bioinformatically predicted open reading frame (ORF) encoding a nitroreductase family deazaflavin-dependent oxidoreductase, sharing the orientation of *murC2*, is present between both genes. The same ORF can also be found between *murG* and *murC2* in the genomes of the *Frankia* strains from cluster-1.

### 3.2. Consensus Amino Acid Sequences of Frankia MurC2 Shows Higher Similarity to MurC from Plants than from other Actinobacteria

**Figure 2:**
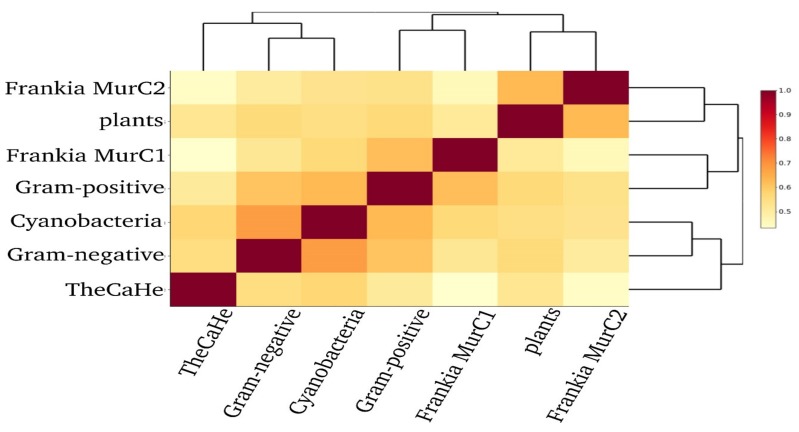
Heatmap and dendrogram showing the sequence similarity of MurC amino acid sequences from *Frankia* (MurC1 and MurC2); plants, Gram-positive bacteria; Gram-negative bacteria; cyanobacteria; and the outliers *Campylobacter, Helicobacter,* and *Thermotoga* (TheCaHe). Light yellow indicates a low sequence similarity, while increasing darker yellow up to red indicates a higher similarity. All of the sequences used are listed in Supplementary Table S2.

The amino acid sequences of MurC from various other species were used to build a maximum-likelihood phylogenetic tree (Supplementary Figure S2). The backbone of the resulting tree allowed for distinguishing the following five different groups: MurC homologs from Gram-positive bacteria (I) in which *Frankia* MurC1 (Ia) proteins are embedded; a branch comprising plant MurC sequences (IIa) and *Frankia* MurC2 (IIb); a branch with the MuC proteins from cyanobacteria (III) and one for Gram-negative bacteria (IV); and last group containing sequences from *Campylobacter, Helicobacter,* and *Thermotoga* (referred to as TheCaHe (V)). Consensus sequences were generated for the different groups using UGENE (alignments are shown in Supplementary Figure S3). A heatmap was generated to visualise the distance similarity matrix of the consensus sequence for each of the groups (Figure 2), as well as for all of the analysed sequences separately (interactive Supplementary Figure S1). The consensus sequence of *Frankia* MurC1 showed the highest similarity with the consensus of MurC from other actinobacteria (62%), while the consensus sequence of *Frankia* MurC2 displayed the highest similarity with the consensus sequence of MurC from plants (63%). The amino acid similarity between the consensus sequences of *Frankia* MurC1 and MurC2 was at 46%, while the similarity of either consensus sequence compared with the consensus sequence of Gram-negative bacteria, cyanobacteria, or CaHeFra, individually, was below 57%. 

### 3.3. No Sequence Evidence for Horizontal Gene Transfer (HGT) could be Found

The similarity of MurC2 with plant MurC proteins suggested the possibility of HGT. Therefore, IslandViewer [36] was used to identify the genomic islands (GIs), commonly defined as clusters of genes of a probable horizontal origin in microbial genomes. However, GC content analysis and tetranucleotide analysis (Supplementary Figure S4) could not confirm the HGT hypothesis. Nevertheless, keeping in mind that if the copy was acquired by HGT, this had to have taken place at least 100 Mya [47], which means sufficient time passed for the codon adaptation of the copy to the genome. In summary, the HGT hypothesis could be neither confirmed nor disproven.

### 3.4. Protein Modelling Reveals Little Structural Difference among Different Groups of MurC Sequences

**Figure 3:**
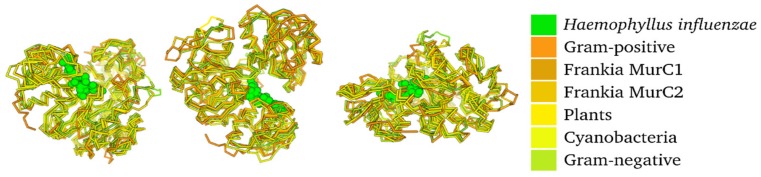
Protein structure model of MurC proteins from Gram-positive bacteria, Gram-negative bacteria, *Frankia* MurC1, and *Frankia* MurC2 (illustrated in various shades of orange and yellow) compared with the solved crystal structure of MurC in *Haemophilus influenzae* (illustrated in green), presented from three different angles.

While the amino acid sequence similarity between the MurC1 and MurC2 proteins was low, the modelling of the proteins revealed little structural differences (Figure 3). Most of the protein models (illustrated in yellow and orange) could be aligned to each other and to the crystal structure of *Haemophilus influenzae* MurC (given in green, Figure 3). The amino acid sequences close to the substrate-binding site showed the highest conservation (given in magenta and indicated by an arrow, Supplementary Figure S5), while the least conservation was found outside of the active sites of the enzyme (illustrated in blue, Supplementary Figure S5). 

### 3.5. Gene Expression Data Shows Differential Expression of murC1 and murC2 in Frankia Cluster-2 in Nodules, but not in Frankia Cluster-3 

**Figure 4:**
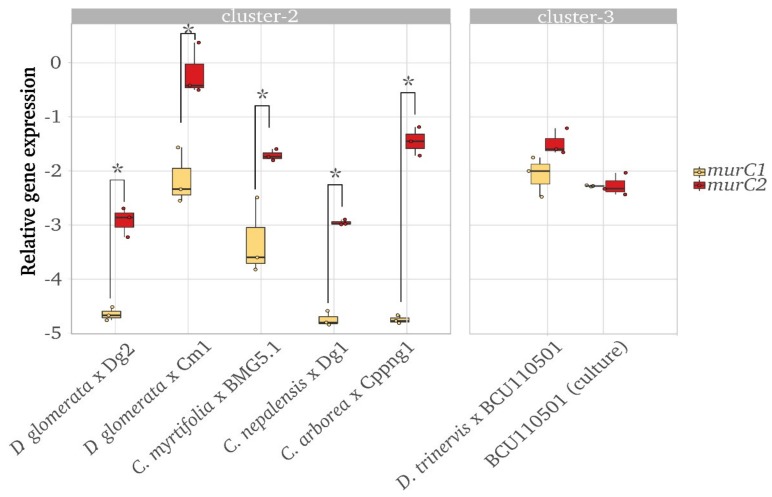
Relative gene expression levels of *murC1 (*yellow) and *murC2* (red) in nodules induced by *Frankia* strains from cluster-2 (left graph; *Candidatus* Frankia californiensis Dg2 [14], *Candidatus* Frankia datiscae Cm1 [14], *Frankia coriariae* BMG5.1 [17], *Candidatus* Frankia datiscae Dg1 [15], and *Candidatus* Frankia meridionalis Cppng1 [13]), and in the nodules induced by the in vitro cultivated strain from cluster-3 (right graph; *Frankia discariae* BCU110501 [22]). The boxplots represent the mean of three independent biological samples; individual data points are presented by dots. The expression levels were determined by RT-qPCR and normalized against the expression level of the translation initiation factor three (IF-3), *infC*. Significant differences between the expression levels of *murC1* and *murC2,* individually, were found for strains from cluster-2 (student’s *t*-test, *p* < 0.05 indicated by asterisk *), but not from cluster-3.

In order to determine whether either *murC1* or *murC2* was preferentially expressed in symbiosis, the relative expression levels of *murC1* and *murC2* were studied in nodules induced by cluster-2 strains; as these strains have not been able to be cultivated thus far, their expression levels under non-symbiotic conditions could not be analysed. For the cluster-3 strain *Frankia discariae* BCU110501, the expression levels of *murC1* and *murC2* were compared in the nodules as well as in nitrogen-fixing in vitro cultures (Figure 4). Overall, *murC2* was found to be expressed at significantly higher levels than *murC1* in the nodules induced by the cluster-2 strains belonging to different uncultured species, i.e., *Candidatus* Frankia datiscae Dg1, *Candidatus* F. datiscae Cm1, *Candidatus* Frankia californiensis Dg2, and *Candidatus* Frankia meridionalis Cppng1, as well as in *Frankia coriariae* BMG5.1 [13,14,15,17,22]. In the only cluster-3 strain examined, *F. discariae* BCU110501, no significant differences between the expression levels of the two genes could be found either in the nodules or in the culture. 

## 4. Discussion

The genomes of the *Frankia* strains from cluster-2 and cluster-3 contain two types of *murC, murC1* and *murC2,* while the genomes of the cluster-1 and cluster-4 strains only contain *murC2.* As the analysis included seven cluster-1 genomes and four cluster-4 genomes, the possibility that *murC1* was missed every time because of insufficient coverage can be excluded. Furthermore, both genes are linked in cluster -1 and cluster-3; so, missing one of them repeatedly would be even more unlikely. Based on the sequence comparisons and phylogenetic analysis, MurC1 is more similar to MurC of other Gram-positive bacteria, including actinobacteria, and should therefore be considered as the ancestral type of *Frankia* MurC. As cluster-2 represents the earliest divergent symbiotic cluster [13,14,15,16,17], it can be concluded that the second type, *murC2*, which is present in all *Frankia* genomes available, was acquired by the common ancestor and maintained in the strains from all four clusters. The phylogeny of MurC2 supports the position of cluster-2 as the earliest divergent cluster, contradicting previous results [15], which can be explained by the lack of differentiation between *murC1* and *murC2* in the earlier analysis. Interestingly, the amino acid sequences of *Frankia* MurC2 shows more similarity to the sequences of plant MurC than to the MurC sequences taken from other bacteria. Similarly, in the phylogenetic tree, *Frankia* MurC2 and plant MurC appear as sister groups. Two scenarios are possible to explain these results. 

First, *murC2* could have been acquired via HGT by the common *Frankia* ancestor from an unidentified source. This should have preceded the evolution of the actinorhizal root nodule symbiosis and the separation between symbiotic and non-symbiotic strains. It is assumed that root nodule symbioses evolved ca. 100 Mya, and that *Frankia* strains were the original microsymbionts [47]. The fact that MurC2 shows a higher similarity to plant than bacterial MurC sequences would be consistent with a close relationship between future host plants, and *Frankia* as a precondition for the evolution of an intracellular symbiosis. However, if *murC2* was acquired from the future plant host, it would be expected to find the highest similarity with angiosperm MurC sequences, which diverged from gymnosperms ca. 200 Mya. Our data do not support this. Recent studies have shown that the plant enzymes for the synthesis of chloroplast peptidoglycan, such as MurC, do not originate from cyanobacteria, but from an unknown bacterial source [22,48]. Thus, the origin of Mur ligase genes is clearly more complex than it was assumed when the endosymbiosis hypothesis first arose. At any rate, using software for the identification of putative genomic islands, the HGT hypothesis could be neither confirmed nor disproven.

The second explanation for the presence of two types of *murC* in *Frankia* genomes is a gene duplication event. This could then have been followed by convergent evolution, which led to more similarity between MurC2 and plant MurC proteins. *Frankia* are able to grow both in soil and intracellularly in the plant during root nodule symbiosis. Within symbiosis, bacterial differentiation is steered by the plant (reviewed by Pawlowski and Demchenko [9]), comparable to the direction of the differentiation of chloroplasts by the host cell. The similarity of *Frankia* MurC2 to plant MurC proteins might be associated with the presence of regulatory processes, which allow for easier structural changes of the peptidoglycan required for the intracellular lifestyle. This assumption is supported by the fact that in cyanobacteria, *murC* and *murB* were found to play a role in the differentiation of nitrogen-fixing heterocysts [7]. However, this would not explain why members of *Frankia* cluster-4, which do not engage in symbiosis, would have retained the MurC type more similar to that of plants over the ancestral MurC type of Gram-positive bacteria. At the same time, cluster-2 strains, which have a limited saprotrophic potential [14], did retain both types. 

Analysis of the expression levels of both *murC* types showed that in the cluster-2 strains, *murC2* was expressed at higher levels than *murC1*. Based on their organisation within the genome (Figure 1), it is possible that a weak terminator is present between the two copies in the cluster-2 genomes, resulting in lower expression levels of *murC1*. Intriguingly, in the nodules induced by a member of *Frankia* cluster-3, as well as in nitrogen-fixing cultures of the same strain, both types of *murC* were expressed at similar levels. However, it is known that MurC activity is regulated post-translationally by a Ser/Thr kinase [49]. Hence, it is not clear whether the transcription levels reflect the enzyme activity levels. 

The synteny of the *Frankia murC* genes (Figure 1) does not seem to support a common origin of *murC2.* The situation in the genomes of the cluster-1, -3, and -4 strain would be consistent with a common origin; the synteny in cluster-3 would then represent the ancestral situation, while the cluster-1 strains would have lost *murC1* and the cluster-4 strains would have lost *murC1* as well as the conserved gene. However, the synteny of *murC1* and *murC2* in the genomes of the cluster-2 strains, while supporting an origin of *murC2* by duplication of *murC1*, does not fit this pattern. In summary, if the similarity between *Frankia murC2* and plant *murC* genes is due to convergent evolution, duplication of *murC,* followed by adaptive evolution of the second copy, could have happened more than once during the evolution of *Frankia.*

Altogether, *Frankia murC1* has been replaced by *Frankia murC2* in cluster-1 and cluster-4, and is expressed at much lower levels than *Frankia murC2* in cluster-2. Thus, selection clearly favours *murC2* over *murC1* in *Frankia.* This could not be linked to differences in the protein structure, so it should be due to differences in the secondary modification.

## 5. Conclusions

*Frankia* genomes can contain two types of *murC, murC1* and *murC2.* The former encodes a protein more similar to the MurC of other Gram-positive bacteria, including actinobacteria, and thus presumably represents the ancestral type of *Frankia* MurC. The presence of *murC2* in the strains of the non-symbiotic cluster-4 indicates that the origin of *murC2* predates the evolution of actinorhizal symbioses. 

MurC2 shows a higher similarity with the MurC proteins from plants than from bacteria, which could be explained by HGT or by gene duplication followed by convergent evolution; detailed phylogenetic analysis involving MurC proteins from different groups of higher plants, as well as the synteny situation in *Frankia* genomes, favour the second option.

In spite of the fact that the amino acid sequences of MurC1 and MurC2 show significant divergence, *murC1* was lost in *Frankia* clusters -1 and -4, and *murC2* shows consistently higher expression levels in symbiosis in *Frankia* cluster-2, indicating selection for *murC2* over *murC1*; modelling did not show any significant structural differences between both types of MurC.

## Supplementary Materials

The following are available online at https://www.mdpi.com/2073-4425/11/4/432/s1. Figure S1: Interactive homology heatmap of all MurC sequences used in this study. Figure S2: ML phylogenetic tree of MurC protein sequences taken from Gram-postive (yellow), *Frankia* (light orange), plants (orange), cyanobacteria (light red), Gram negative (red), and *Campylobacter, Helicobacter,* and *Thermotoga (*abbreviated TheCaHe, purple) sequences, with support values of 250 bootstrap iterations. Figure S3: (**a**) Alignment of all MurC sequences to obtain the consensus sequences of Gram Positives, *Frankia* MurC1, Plants, *Frankia* MurC2, Cyanobacteria, Gram-Negatives and the TheCaHe group, using the CLUSTAL Omega algorithm with 25 iterations, trimmed using UGene. (**b**) The consensus sequences for the different groups determined at 75% similarity threshold using UGene, presented using JaIview (Waterhouse, A.M.; Procter, J.B.; Martin, D.M.A; Clamp, M.; Barton, G.J. Jalview Version 2 - a multiple sequence alignment editor and analysis workbench. *Bioinformatics* 2009, *25,* 1189–1191. Figure S4: Tetranucleotide analysis for the identification of genomic islands (GIs). IslandViewer 4 was used to identify GIs in different *Frankia* genomes [36]. Predictions of genomic islands in the *Candidatus* Frankia datiscae Dg1 genome (accession number CP002801.1) are shown in a circular visualization, with blocks coloured according to the prediction method: IslandPick (green), IslandPath-DIMOB (blue), SIGI-HMM (orange), Islander (turquoise). Figure S5: Models of MurC protein of Gram positive bacteria, Gram negative bacteria, cyanobacteria, *Frankia,* and plants, using the solved crystal structure of *Haemophilus influenzae* as template (PDB accession number: 1P31), using Consurf software. Conserved sites are illustrated in magenta, less conserved sites in blue. Table S1: Primers used in this study. Table S2: *MurC* protein sequences used in this study.

## References

[1] Gantt E., Bryant D.A. (1994). Supramolecular membrane organization. The Molecular Biology of Cyanobacteria.

[2] Takano H., Takechi K. (2010). Plastid peptidoglycan. Biochim. Biophys. Acta..

[3] Vollmer W., Seligman S.J. (2009). Architecture of peptidoglycan: More data and more models. Trends Microbiol..

[4] Sato N., Toyoshima M., Tajima N., Takechi K., Takano H. (2017). Single-pixel densitometry revealed the presence of peptidoglycan in the intermembrane space of the moss chloroplast envelope in conventional electron micrographs. Plant Cell Physiol..

[5] Barreteau H., Kovač A., Boniface A., Sova M., Gobec S., Blanot D. (2008). Cytoplasmic steps of peptidoglycan biosynthesis. FEMS Microbiol. Rev..

[6] Munshi T., Gupta A., Evangelopoulos D., Guzman J.D., Gibbons S., Keep N.H., Bhakta S. (2013). Characterisation of ATP-dependent mur ligases involved in the biogenesis of cell wall peptidoglycan in *Mycobacterium tuberculosis*. PLoS ONE.

[7] Videau P., Rivers O.S., Ushijima B., Oshiro R.T., Kim M.J., Philmus B., Cozy L.M. (2016). Mutation of the *murC* and *murB* genes impairs heterocyst differentiation in *Anabaena* sp. strain PCC 7120. J. Bacteriol..

[8] Garcia M., Myouga F., Takechi K., Sato H., Nabeshima K., Nagata N., Takio S., Shinozaki K., Takano H. (2008). An Arabidopsis homolog of the bacterial peptidoglycan synthesis enzyme MurE has an essential role in chloroplast development. Cell. Mol. Biol..

[9] Pawlowski K., Demchenko K.N. (2012). The diversity of actinorhizal symbiosis. Protoplasma.

[10] Normand P., Orso S., Cournoyer B., Jeannin P., Chapelon C., Dawson J., Evtushenko L., Misra A.K. (1996). Molecular phylogeny of the genus *Frankia* and related genera and emendation of the family Frankiaceae. Int. J. Syst. Bacteriol..

[11] Clawson M.L., Bourret A., Benson D.R. (2004). Assessing the phylogeny of *Frankia-*actinorhizal plant nitrogen-fixing root nodule symbiosis with *Frankia* 16S rRNA and glutamine synthetase gene sequences. Mol. Phylogenetics Evol..

[12] Maréchal J., Clement B., Nalin R., Gandon C., Orso S., Cvejic J.H., Bruneteau M., Berry A.M., Normand P. (2000). A *recA* gene phylogenetic analysis confirms the close proximity of *Frankia* to *Acidothermus*. Int. J. Syst. Evol. Microbiol..

[13] Nguyen T.V., Wibberg D., Battenberg K., Blom J., Vanden Heuvel B., Berry A.M., Kalinowski J., Pawlowski K. (2016). An assemblage of *Frankia* cluster II strains from California contains the canonical *nod* genes and also the sulfotransferase gene *nodH*. BMC Genomics..

[14] Nguyen T.V., Wibberg D., Vigil-Stenman T., Berckx F., Battenberg K., Demchenko K., Blom J., Fernandez M.P., Yamanaka T., Berry A.M. (2019). *Frankia*-enriched metagenomes from the earliest divergent symbiotic *Frankia* cluster: They come in teams. Genome Biol. Evol..

[15] Persson T., Battenberg K., Demina I.V., Vigil-Stenman T., Vanden Heuvel B., Pujic P., Facciotti M.T., Wilbanks E.G., O’Brien A., Fournier P. (2015). *Candidatus* Frankia Datiscae Dg1, the actinobacterial microsymbiont of *Datisca glomerata*, expresses the canonical *nod* genes *nodABC* in symbiosis with its host plant. PLoS ONE.

[16] Pozzi A.C., Bautista-Guerrero H.H., Abby S.S., Herrera-Belaroussi A., Abrouk D., Normand P., Menu F., Fernandez M.P. (2018). Robust *Frankia* phylogeny, species delineation and intraspecies diversity based on Multi-Locus Sequence Analysis (MLSA) and Single-Locus Strain Typing (SLST) adapted to a large sample size. Sys. App. Microbiol..

[17] Gtari M., Ghodhbane-Gtari F., Nouioui I., Ktari A., Hezbri K., Mimouni W., Sbissi I., Ayari A., Yamanaka T., Normand P. (2015). Cultivating the uncultured: Growing the recalcitrant cluster-2 *Frankia* strains. Sci. Rep..

[18] Gueddou A., Swanson E., Hezbri K., Nouioui I., Ktari A., Simpson S., Morris K., Kelley Thomas W., Ghodhbane-Gtari F., Gtari M. (2019). Draft genome sequence of the symbiotic *Frankia* sp. strain BMG5.30 isolated from root nodules of *Coriaria myrtifolia* in Tunisia. Antonie Van Leeuwenhoek.

[19] Hurles M. (2004). Gene duplication: The genomic trade in spare parts. PLoS Biol..

[20] Sato N. (2020). Complex origins of chloroplast membranes with photosynthetic machineries: Multiple transfers of genes from divergent organisms at different times or a single endosymbiotic event?. J. Plant Res..

[21] Normand P., Nguyen T.V., Battenberg K., Berry A.M., van den Heuvel B., Fernandez M.P., Pawlowski K. (2017). Proposal of ‘*Candidatus* Frankia californiensis’, the uncultured symbiont in nitrogen-fixing root nodules of a phylogenetically broad group of hosts endemic to western North America. Int. J. Syst. Evol. Microbiol..

[22] Nouioui I., Del Carmen Montero-Calasanz M., Ghodhbane-Gtari F., Rohde M., Tisa L.S., Klenk H.P., Gtari M. (2017). *Frankia discariae* sp. nov.: An infective and effective microsymbiont isolated from the root nodule of Discaria trinervis. Arch Microbiol..

[23] Hoagland D.R., Arnon D.I. (1938). The Water-Culture Method for Growing Plants without Soil.

[24] Schwencke J. (1991). Rapid, exponential growth and increased biomass yield of some *Frankia* strains in buffered and stirred mineral medium (BAP) with phosphatidyl choline. Plant Soil..

[25] Benchling [Biology Software]. https://benchling.com.

[26] Alloisio N., Queiroux C., Fournier P., Pujic P., Normand P., Vallenet D., Médigue C., Yamaura M., Kakoi K., Kucho K.-I. (2010). The *Frankia alni* symbiotic transcriptome. Mol. Plant Microbe Interact..

[27] RStudio Team (2015). Rstudio: Integrated Development.

[28] Meyer F., Goesmann A., McHardy A.C., Bartels D., Bekel T., Clausen J., Kalinowski J., Linke B., Rupp O., Giegerich R. (2003). GenDB--an Open Source Genome Annotation System for prokaryote genomes. Nucleic Acids Res..

[29] Markowitz V.M., Chen I.A., Palaniappan K., Chu K., Szeto E., Grechkin Y., Ratner A., Jacob B., Huang J., Williams P. (2012). IMG: The Integrated Microbial Genomes Database and Comparative Analysis System. Nucleic Acids Res.

[30] Wong G.K., Soltis D.E., Leebens-Mack J., Wickett N.J., Barker M.S., Van de Peer Y., Graham S.W., Melkonian M. (2019). Sequencing and analyzing the transcriptomes of a thousand species across the tree of life for green plants. Annu. Rev. Plant Biol..

[31] Carpenter E.J., Matasci N., Ayyampalayam S., Wu S., Sun J., Yu J., Jimenez Vieira F.R., Bowler C., Dorrell R.G., Gitzendanner M.A. (2019). Access to RNA-sequencing data from 1,173 plant species: The 1000 Plant transcriptomes initiative (1KP). GigaScience.

[32] One Thousand Plant Transcriptomes Initiative (2019). One thousand plant transcriptomes and the phylogenomics of green plants. Nature.

[33] Okonechnikov K., Golosova O., Fursov M. (2012). The UGENE team. Unipro UGENE: A unified bioinformatics toolkit. Bioinformatics.

[34] Sievers F., Wilm A., Dineen D., Gibson T.J., Karplus K., Li W., Lopez R., McWilliam H., Remmert M., Söding J. (2011). Fast, scalable generation of high-quality protein multiple sequence alignments using Clustal Omega. Mol. Syst. Biol..

[35] Miller M.A., Pfeiffer W., Schwartz T. Creating the CIPRES science gateway for inference of large phylogenetic trees. Proceedings of the Gateway Computing Environments Workshop (GCE).

[36] Bertelli C., Laird M.R., Williams K.P., Lau B.Y., Hoad G., Winsor G.L., Brinkman F.S.L., Simon Fraser University Research Computing Group (2017). IslandViewer 4: Expanded prediction of genomic islands for larger-scale datasets. Nucleic Acids Res..

[37] Benkert P., Biasini M., Schwede T. (2011). Toward the estimation of the absolute quality of individual protein structure models. Bioinformatics.

[38] Bertoni M., Kiefer F., Biasini M., Bordoli L., Schwede T. (2017). Modeling protein quaternary structure of homo- and hetero-oligomers beyond binary interactions by homology. Sci. Rep..

[39] Bienert S., Waterhouse A., de Beer T.A.P., Tauriello G., Studer G., Bordoli L., Schwede T. (2017). The SWISS-MODEL repository-new features and functionality. Nucleic Acids Res..

[40] Guex N., Peitsch M.C., Schwede T. (2009). Automated comparative protein structure modeling with SWISS-MODEL and Swiss-PdbViewer: A historical perspective. Electrophoresis.

[41] Waterhouse A., Bertoni M., Bienert S., Studer G., Tauriello G., Gumienny R., Heer F.T., de Beer T.A.P., Rempfer C., Bordoli L. (2018). SWISS-MODEL: Homology modelling of protein structures and complexes. Nucleic Acids Res..

[42] Holm L. (2019). Benchmarking fold detection by DaliLite v.5. Bioinformatics.

[43] Mol D.C., Brooun A., Dougan D.R., Hilgers M.T., Tari L.W., Wijnands R.A., Knuth M.W., McRee D.E., Swanson R.V. (2003). Crystal structures of active fully assembled substrate- and product-bound complexes of UDP-N-acetylmuramic acid:L-alanine ligase (MurC) from *Haemophilus influenzae*. J. Bacteriol..

[44] Ashkenazy H., Abadi S., Martz E., Chay O., Mayrose I., Pupko T., Ben-Tal N. (2016). ConSurf 2016: An improved methodology to estimate and visualize evolutionary conservation in macromolecules. Nucleic Acids Res..

[45] Ashkenazy H., Erez E., Martz E., Pupko T., Ben-Tal N. (2010). ConSurf 2010: Calculating evolutionary conservation in sequence and structure of proteins and nucleic acids. Nucleic Acids Res..

[46] Celniker G., Nimrod G., Ashkenazy H., Glaser F., Martz E., Mayrose I., Pupko T., Ben-Tal N. (2013). ConSurf: Using evolutionary data to raise testable hypotheses about protein function. ISR J Chem..

[47] Van Velzen R., Doyle J.J., Geurts R. (2019). A resurrected scenario: Single gain and massive loss of nitrogen-fixing nodulation. Trends Plant Sci..

[48] Sato N., Takano H. (2017). Diverse origins of enzymes involved in the biosynthesis of chloroplast peptidoglycan. Plant Res..

[49] Falk S.P., Weisblum B. (2013). Phosphorylation of the *Streptococcus pneumoniae* cell wall biosynthesis enzyme MurC by a eukaryotic-like Ser/Thr kinase. FEMS Microbiol. Lett..

