# Supplementary material for: The Peptidoglycan Biosynthesis Gene murC in Frankia: Actinorhizal vs. Plant Type
Source: Genes (Basel). 2020 Apr 16;11(4):432. doi: 10.3390/genes11040432 (PMC7231273; doi:10.3390/genes11040432)
Supplement: Supplementary file 1 [file genes-11-00432-s001.zip › Supplementary/Supplementary Figure_S1_full_heatmap.html]

Untitled


# Untitled

## R Markdown

This is an R Markdown document. Markdown is a simple formatting syntax for authoring HTML, PDF, and MS Word documents. For more details on using R Markdown see http://rmarkdown.rstudio.com.

When you click the **Knit** button a document will be generated that includes both content as well as the output of any embedded R code chunks within the document. You can embed an R code chunk like this:

## Including Plots

You can also embed plots, for example:

```
## [1] 0.3152941 1.0000000
```

```
## Warning: 'heatmap' objects don't have these attributes: 'showlegend'
## Valid attributes include:
## 'type', 'visible', 'opacity', 'name', 'uid', 'ids', 'customdata', 'meta', 'hoverinfo', 'hoverlabel', 'stream', 'transforms', 'uirevision', 'z', 'x', 'x0', 'dx', 'y', 'y0', 'dy', 'text', 'hovertext', 'transpose', 'xtype', 'ytype', 'zsmooth', 'connectgaps', 'xgap', 'ygap', 'zhoverformat', 'hovertemplate', 'zauto', 'zmin', 'zmax', 'zmid', 'colorscale', 'autocolorscale', 'reversescale', 'showscale', 'colorbar', 'coloraxis', 'xcalendar', 'ycalendar', 'xaxis', 'yaxis', 'idssrc', 'customdatasrc', 'metasrc', 'hoverinfosrc', 'zsrc', 'xsrc', 'ysrc', 'textsrc', 'hovertextsrc', 'hovertemplatesrc', 'key', 'set', 'frame', 'transforms', '_isNestedKey', '_isSimpleKey', '_isGraticule', '_bbox'
```

Note that the `echo = FALSE` parameter was added to the code chunk to prevent printing of the R code that generated the plot.
