# Supplementary figures and images for: The Peptidoglycan Biosynthesis Gene murC in Frankia: Actinorhizal vs. Plant Type
Source: Genes (Basel). 2020 Apr 16;11(4):432. doi: 10.3390/genes11040432 (PMC7231273; doi:10.3390/genes11040432)

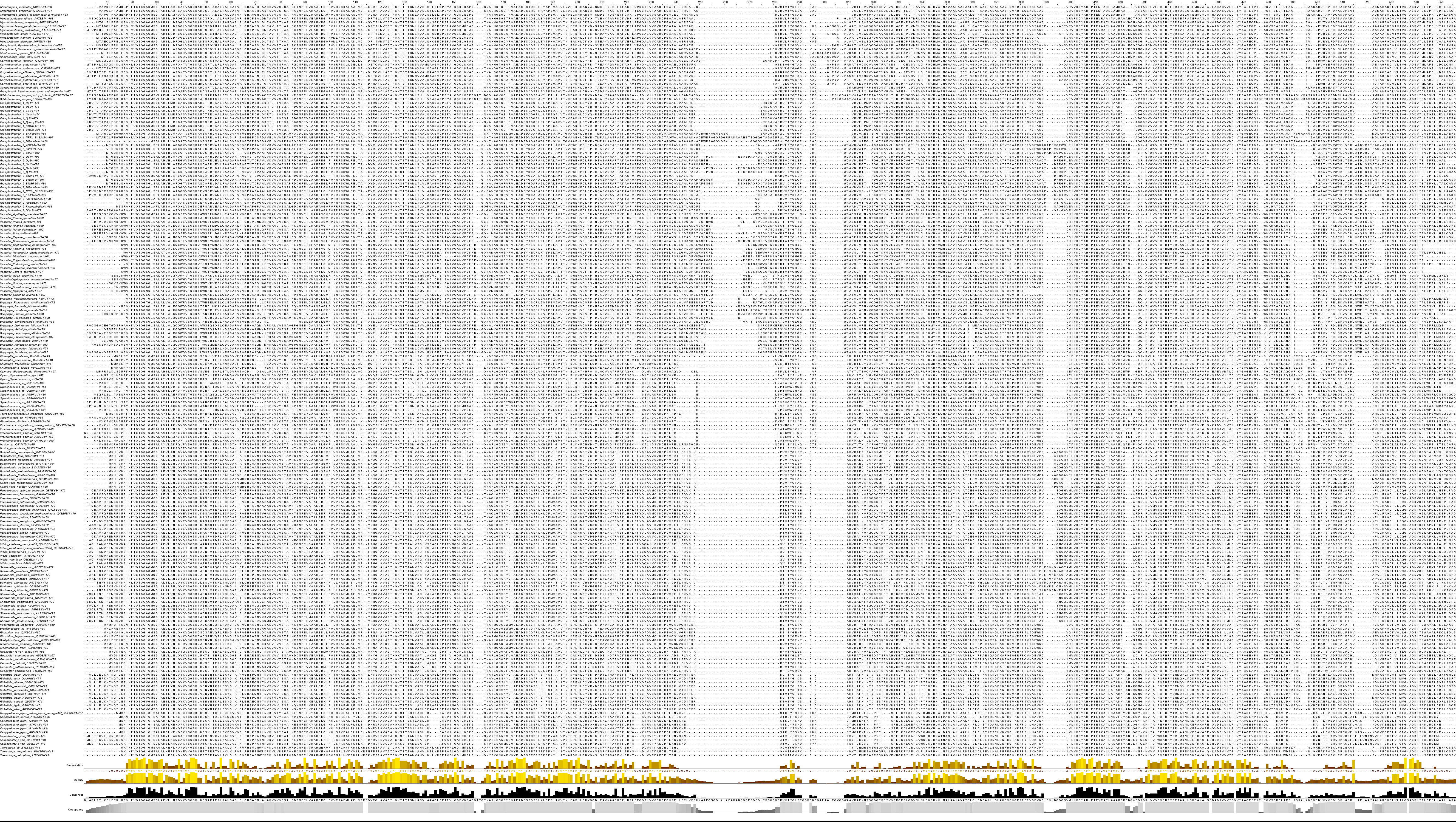

Supplement: Supplementary file 1 [file genes-11-00432-s001.zip › Supplementary/Supplementary Figure_S3a.jpg]

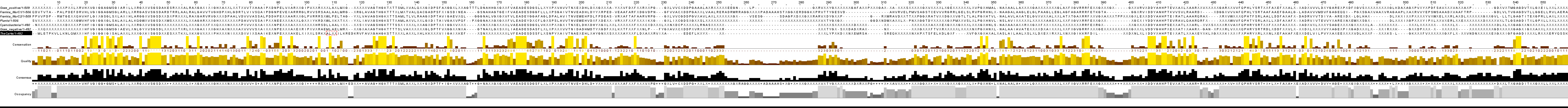

Supplement: Supplementary file 1 [file genes-11-00432-s001.zip › Supplementary/Supplementary Figure_S3b.jpg]

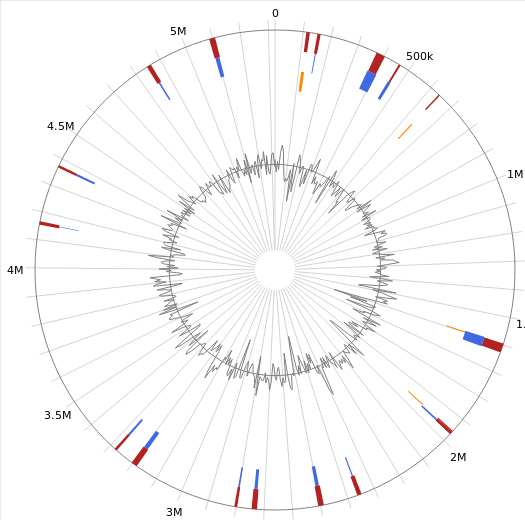

Supplement: Supplementary file 1 [file genes-11-00432-s001.zip › Supplementary/Supplementary Figure_S4_tetranucleotide_analysis.jpg]

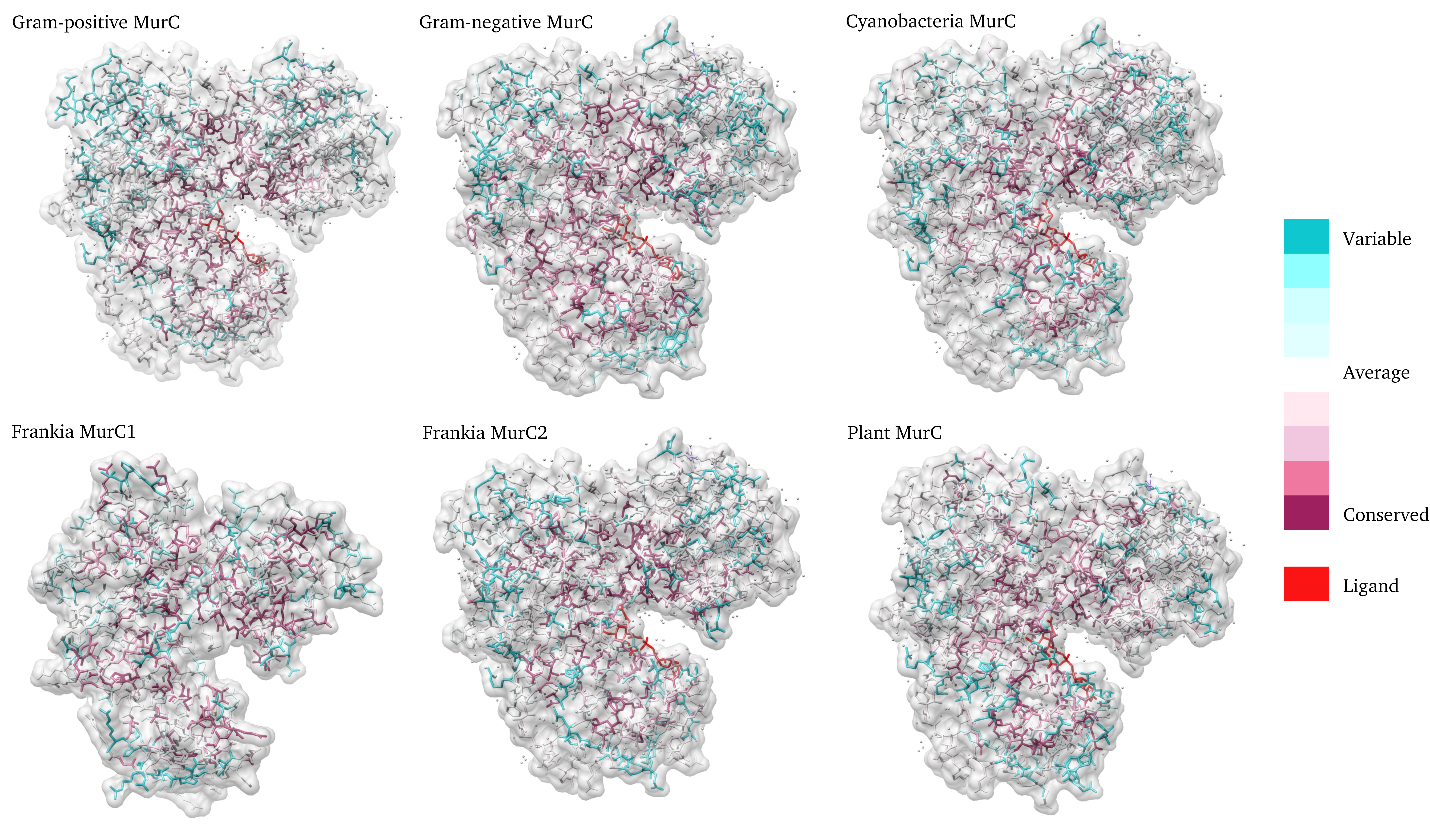

Supplement: Supplementary file 1 [file genes-11-00432-s001.zip › Supplementary/Supplementary Figure_S5_protein modeling with scale.jpg]
